# Effects of the Extracts from Fruit and Stem of *Camellia japonica* on Induced Pluripotency and Wound Healing

**DOI:** 10.3390/jcm7110449

**Published:** 2018-11-20

**Authors:** Hyejin Jeon, Jae Yun Kim, Jung‐Kyun Choi, Enna Han, Cho-Lok Song, Jungwoon Lee, Yee Sook Cho

**Affiliations:** 1 Stem Cell Research Laboratory, Immunotherapy Convergence Research Center, Korea Research Institute of Bioscience and Biotechnology (KRIBB), 125 Gwahak-ro, Yuseong-gu, Daejeon 34141, Korea; hjjeon@kribb.re.kr (H.J.); jameskim@kribb.re.kr (J.Y.K.); cjk2916@kribb.re.kr (J.-K.C.); enh9321@kribb.re.kr (E.H.); clsong@kribb.re.kr (C.-L.S.); 2 Department of Bioscience, KRIBB School, University of Science and Technology (UST), 113 Gwahak-ro, Yuseong-gu, Daejeon 34113, Korea

**Keywords:** plant extracts, reprogramming, induced pluripotent stem cell, wound healing

## Abstract

Small molecules that improve reprogramming, stem cell properties, and regeneration can be widely applied in regenerative medicine. Natural plant extracts represent an abundant and valuable source of bioactive small molecules for drug discovery. Natural products themselves or direct derivatives of them have continued to provide small molecules that have entered clinical trials, such as anticancer and antimicrobial drugs. Here, we tested 3695 extracts from native plants to examine whether they can improve induced pluripotent stem cell (iPSC) generation using genetically homogeneous secondary mouse embryonic fibroblasts (MEFs) harboring doxycycline (dox)-inducible reprograming transgenes. Among the tested extracts, extracts from the fruit and stem of *Camellia japonica* (*CJ*) enhanced mouse and human iPSC generation and promoted efficient wound healing in an in vivo mouse wound model. *CJ* is one of the best-known species of the genus *Camellia* that belongs to the Theaceae family. Our findings identified the natural plant extracts from the fruit and stem of *CJ* as novel regulators capable of enhancing cellular reprogramming and wound healing, providing a useful supplement in the development of a more efficient and safer method to produce clinical-grade iPSCs and therapeutics.

## 1. Introduction

Tissue regeneration in multicellular organisms requires cellular reprogramming processes, involving developmental programs for wound healing, apoptosis, differentiation, and proliferation [1,2,3,4,5]. Aging and the accumulation of damage, including DNA, protein, and lipid damage from oxidative stress, are associated with degenerative diseases, such as wound healing disorders, cancer, cardiovascular disease, immune disease, and dementia [6]. Hence, there have been many efforts to develop regenerative technologies associated with reprogramming process that rejuvenate old cells into younger cells. Somatic cellular reprogramming into pluripotency is associated with rejuvenation, demonstrating the possibility to reverse aging. Characteristic features of a rejuvenation process, such as reorganized mitochondria, reduced oxidative stress, and elongated telomeres, are also similar to those observed during the reprogramming process of aged somatic cells to a youthful state [7,8]. For example, oxidative damage not only accumulates with age but also causes aging and interferes with the reprogramming process. Thus, antioxidants, such as N-acetyl-cysteine (NAC) or vitamin C (Vc), prevent this damage and significantly enhance reprogramming efficiency [9]. Thus, research on a variety of pharmacological approaches to improve reprogramming efficiency and tissue regeneration is necessary for stem-cell-related regenerative medicine.

The generation of induced pluripotent stem cells (iPSCs) by somatic cellular reprogramming technology using a combination of defined reprogramming factors termed the “Yamanaka factors” (Oct-4, Sox-2, Klf-4, and c-Myc (OSKM)) represents a major breakthrough in stem cell biology and regenerative medicine [10,11]. Personalized human iPSCs (hiPSCs), which can be grown as 3D architecture-organoids in a dish mimicking in vivo tissues, are expected to contribute to applications in the fields of human disease modeling, translational medicine, and personalized therapy [12]. Considerable efforts have been made to improve the efficiency and safety of somatic cellular reprogramming to generate genetically unmodified hiPSCs for clinical applications. Various small molecules, including epigenetic modifiers, metabolic regulators, and antioxidants, and herbal decoction Sagunja-Tang, have been identified that are involved in the reprogramming process and promote the efficiency of iPSC generation [13,14,15,16]. In particular, chemically induced iPSCs (CiPSCs) can be generated from mouse somatic cells using a combination of seven small molecules, including VC6T (VPA, CHIR99021 a GSK inhibitor, 616452 and Tranylcypromine a monoamine oxidase inhibitor) plus forskolin (a cAMP agonist) and 3-deazaneplanocin A (DZNep, a global histone methylation inhibitor) but not from human cells [17]. Therefore, more efforts are required to identify small molecules involved in human somatic cellular reprogramming. 

Natural products, such as plant extracts, provide unlimited resources for drug discovery given the outstanding availability of chemical diversity [18,19]. Plant extracts have been used worldwide for thousands of years as traditional medicines for treatment of various human diseases, and the effectiveness of bioactive compounds from plant extracts are clinically validated [20,21]. Different beneficial biological activities, such as anticancer [22,23], antimicrobial [24], antioxidant [25,26], antiaging [27,28], antianalgesic [29], and wound healing activities [30,31], have been reported. Furthermore, unlike most animals, plants cells are thought to be totipotent (i.e., regenerate completely new plant tissues cultured in vitro [4]) or pluripotent (i.e., regenerate some plant tissues with restricted potential [1,32]), suggesting that plant extracts are expected to contain the valuable bioactive small molecules involved in reprogramming, rejuvenation, and/or regeneration processes. 

Previous studies have demonstrated that *Camellia japonica* (*CJ*), one of the best known species of the genus *Camellia* that belongs to the Theaceae family and is widely grown in Korea and Japan [33], has a variety of biological activities, including antioxidant [33,34], anti-inflammatory [35], antimetastasis [36], antiallergic responses [37], antibacterial activity [38], antiatherosclerosis [39], antiatherogenic [40], and antiphotoaging activities [41]. Generally, extensive research on the pharmacological activity of *CJ* has been studied in the plant’s oil, flower, or leaf [39]. Interestingly, the extracts of *CJ* fruits exhibit a strong cardiovascular protective effect, inducing endothelium-dependent nitric oxide (NO)-mediated relaxation via the redox-sensitive PI3-kinase pathway [39]. In addition, anti-inflammatory and gastroprotective mechanisms of *CJ* fruits are mediated by modulation of oxidative stress, inflammatory cytokines, and enzymes via suppression of mitogen-activated protein kinases (MAPK)/nuclear factor kappa-light-chain-enhancer of activated B cells (NF-κB) signaling pathways [42]. 

Here, we have focused on the determination of the effect of plant extracts from the fruit and/or stem of *CJ* on the somatic cellular reprogramming process using a comparative cell-based high-throughput screening strategy employing genetically homogeneous secondary mouse embryonic fibroblasts (MEFs) harboring doxycycline (dox)-inducible OSKM transgenes. Our results demonstrate that a combination of the extracts from the fruit and stem of *CJ* can significantly improve hiPSC generation as well as wound healing. Our findings provide evidence that the natural plant extracts from the fruit and stem of *CJ* are potential sources for regenerative medicine and may offer new perspectives for therapeutic strategies.

## 2. Experimental Section

### 2.1. Preparation of Plant Extracts 

A total of 3695 plant extracts were purchased from the Korea Plant Extract Bank at Korea Research Institute of Bioscience & Biotechnology (KRIBB) (http://extract.kribb.re.kr, Ochang, Chungbuk, Korea) [43]. According to the supplier [25,44], each plant material was washed, air-dried at 70 °C, and ground. The dried ground materials were extracted in methyl alcohol (99.9%) at 45 °C for 3 days. The extract solution was then filtered and vacuum-evaporated to dryness. The freeze-dried methanol extracts of each plant (20 mg of each sample) were obtained and dissolved in dimethyl sulfoxide (DMSO; Sigma-Aldrich, St. Louis, MO, USA) at 100 mg/mL and stored as a stock vial at −80 °C. Before use, 100 mg/mL stock solution was diluted to 25 mg/mL with DMSO through stepwise dilution. 

### 2.2. Cell Culture

CRL2097 (human newborn foreskin fibroblasts; ATCC, Rockville, MD, USA) were cultured in MEM (#11095, Invitrogen, Carlsbad, CA, USA) supplemented with 10% FBS (Invitrogen), 1% MEM NEAA (#11140, Invitrogen), 1% P/S (#15140, Invitrogen), and 1% sodium pyruvate (#11360, Invitrogen). Media were changed every other day. Human iPSCs (passage 13–17) were maintained on irradiated MEF feeder cells in Dulbecco’s modified Eagle’s medium/F12 medium (Invitrogen) supplemented with 20% Knockout Serum replacement (Invitrogen), 100 units/mL penicillin and 100 µg/mL streptomycin (Invitrogen), 1% nonessential amino acids (NEAA) (Invitrogen), 1 mM L-glutamine (Invitrogen), 0.1 mM β-mercaptoethanol (Sigma-Aldrich), and 6 ng/mL of basic fibroblast growth factor (bFGF) (R&D Systems, Minneapolis, MN, USA), as previously described [45,46]. Mouse PSCs were cultured in DMEM-high glucose supplemented with 15% FBS, 100 units/mL penicillin, 100 µg/mL streptomycin, 1% NEAA, 1% sodium pyruvate, 0.1 mM β-mercaptoethanol, and 1000 U/mL leukemia inhibitory factor (LIF) (Millipore, Billerica, MA, USA). 

### 2.3. Mice

C57BL/6J and R26^rtTA^; Col1a1^4F2A^ mice were obtained from Jackson Laboratory (Bar Harbor, ME, USA) and maintained at KRIBB. All mice were maintained under specific pathogen-free (SPF) conditions and housed in a standard temperature (20–22 °C), humidity-controlled (50%–60%) environment with a 12 h light/dark cycle with free access to food and water. All animal experiments were approved by the Institutional Animal Care and Use Committee of KRIBB (KRIBB-AEC-18041).

### 2.4. High-Throughput Screening

Following the protocol, *4F2A* MEFs were isolated from embryonic day 13.5 (E13.5) embryos of the single dox-inducible transgenic mouse strains that express four defined genes (*OSKM*) separated by three sequences encoding 2A self-cleaving peptides from the *Col1a1* locus [47]. Briefly, internal organs and heads of embryos were removed before MEF isolation, and then MEFs were expanded in DMEM-high glucose (Invitrogen) supplemented with 10% FBS, 100 units/mL penicillin, 100 µg/mL streptomycin, 1% NEAA, and 0.1 mM β-mercaptoethanol. The reprogramming scheme is consistent with published procedures [47,48,49]. 

### 2.5. Plant-Extract-Induced hiPSC Generation

CRL2097 were seeded at 1 × 10^6^ per well in a six-well plate 1 day before transduction with CytoTune®-iPS 2.0 Sendai Reprogramming Kit (#A16157, Invitrogen, Carlsbad, CA, USA) according to the protocol. At day 5 after transduction, the cells were trypsinized and reseeded on Matrigel-treated (BD Biosciences, San Jose, CA) 12-well plates as previously reported [47]. The plant extracts were added to the culture mTeSR1 (STEMCELL Technologies, Cambridge, MA, USA) medium. The media were changed every other day, and the plant extract was re-added after each media change. To establish stable plant-extract-induced hiPSC lines, single ESC-like colonies were selected, seeded into individual wells of a four-well plate, and expanded on MEF feeder layers for characterization as previously reported [47].

### 2.6. Immunocytochemistry 

The iPSCs were fixed in 4% paraformaldehyde for 15 min. After washing with PBS, the cells were permeabilized in 1% bovine serum albumin (BSA) and 0.1% Triton X-100 for 30 min at room temperature as previously reported [47]. The following primary antibodies were used: Oct4 (1:100, sc-9081, Santa-Cruz, Dallas, TX), Nanog (1:40, AF1997, R&D systems), TRA-1-81 (1:100, MAB4381, Millipore), SSEA3 (1:50, MAB1434, R&D), TRA-1-60 (1:100, MAB4360, Millipore), and SSEA4 (1:30, MAB1435, R&D). The following secondary antibodies were used: chicken anti-rabbit IgG 594 (1:200, A21442, Invitrogen), chicken anti-goat IgG 488 (1:200, A21467, Invitrogen), goat anti-mouse IgM 594 (1:200, A21044, Invitrogen), mouse anti-rat IgM FITC (1:200, #553887, BD Biosciences), and donkey anti-mouse IgG 488 (1:200, A21202, Invitrogen).

### 2.7. In Vitro Differentiation

For embryoid body (EB) formation, we followed the previously reported protocols [47,50]. Briefly, iPSCs were transferred onto nonadherent plates and maintained for 7 days. EBs were plated on the Matrigel-coated glass slides. Ten days later, immunocytochemistry was performed as described above. The following primary antibodies were used: Tuj1 (1:500, PRB-435P, Covance, Princeton, NJ, USA), Nestin (1:100, MAB5326, Millipore), α-SMA (1:200, A5228, Sigma-Aldrich), FOXA2 (1:100, 07-633, Millipore), and Sox17 (1:50, MAB1924, R&D Systems). Secondary antibodies included chicken anti-rabbit IgG 594 (A21442, Invitrogen) and donkey anti-mouse IgG 488 (A21202, Invitrogen).

### 2.8. Teratoma Formation and Histological Analysis

The iPSCs were detached and injected subcutaneously into the dorsal flanks of nude mice as previously reported [47]. Eight weeks after the injection, the teratoma was surgically dissected, fixed, and embedded in paraffin. Sections were stained with hematoxylin and eosin.

### 2.9. Wound Healing Analysis

To evaluate the effects of plant extracts in wound healing assays, an excision wound model was used. Mice in the excision wound healing model were divided into four groups as follows: group I, DMSO-treated or untreated mice considered as control; group II, treated with 2 mg/mL plant extracts; group III, treated with 10 mg/mL plant extract; and group IV, treated with 50 mg/mL plant extracts. All the treatments were administered every other day. Mice from each group were shaved on the dorsal side using depilatory cream (Reckitt Benckiser, UK) and anaesthetized via a 2.5% 2,2,2-Tribromoethanol (Sigma‑Aldrich) intraperitoneal injection. To create the circular wound, a 5-mm biopsy punch (Integra Miltex, Davies Dr, York, PA, USA) was obtained to generate the circular pattern of the wound on each side. Plant extracts were applied once every other day for 12 days. The size of the wound area in each group was measured 0, 3, 5, 7, 10, and 12 days postsurgery.

### 2.10. Histopathological Analysis

For histological examination, mice were sacrificed at 0, 3, 5, 7, 10, and 12 days postsurgery. All the mice were anesthetized using 2.5% 2,2,2-Tribromoethanol (Sigma‑Aldrich). The wound skin tissue samples were fixed overnight in 10% neutral buffered formalin followed by paraffin embedding. The wound tissue samples were cut into 10-μm thick sections from the middle of the wound area and then stained with hematoxylin and eosin for histological analysis.

### 2.11. RNA Isolation and Quantitative Real-Time Reverse Transcriptase PCR

Total RNA was isolated from wound skin tissues using an RNeasy Mini Kit (Qiagen, Valencia, CA, USA) following the manufacturer’s instructions. Complementary DNA (cDNA) was synthesized from 1.5 µg of total RNA using the SuperScript^®^ VILO cDNA Synthesis Kit and Master Mix (Invitrogen). Quantitative real-time reverse transcriptase PCR (qPCR) was performed using Fast SYBR Green Master Mix (Applied Biosystems, Waltham, MA, USA) and a 7500 Fast Real-Time PCR System (Applied Biosystems). The specific primer sequences were previously described [47] and as follows: mGAPDH forward, 5′-AGGTCGGTGTGAACGGATTTG-3′; mGAPDH reverse, 5′-GGGGTCGTTGATGGCAACA-3′; mVEGF-α forward, 5′-GCACATAGAGAGAATGAGCTTCC-3′; mVEGF-α reverse, 5′-CTCCGCTCTGAACA AGGCT-3′.

### 2.12. Western Blot Analysis

Wounded mouse skin tissues were homogenized using the Precellys 24 Tissue Homogenizer (Bertin Instruments, France) in modified RIPA buffer (150 mM Sodium chloride, 1% Triton X-100, 1% sodium deoxycholate, 0.1% SDS, 50 mM Tris-HCl, pH 7.5, and 2 mM EDTA, sterile solution) (#R2002, Biosesang, Korea) containing protease inhibitor cocktail (#P3100, GenDEPOT, Katy, TX, USA) and phosphatase inhibitors cocktail (#P3200, GenDEPOT), using the manufacturer’s instructions. The Western blot analysis was performed as previously described [47]. Tissue lysates were separated by sodium dodecyl sulfate polyacrylamide gel electrophoresis (SDS-PAGE) of 15% gradient and transferred to polyvinylidene difluoride (PVDF) membrane. The membranes were immunoblotted using a vascular endothelial growth factor (VEGF) antibody (1:1000, #MA5-13182, Thermo Fisher, Waltham, MA, USA) or β-actin (1:5000, #SC-47778, Santa Cruz, Dallas, TX, USA). The secondary antibody used was a goat anti-mouse IgG-HRP (#SC-2005, Santa Cruz, CA, USA). 

## 3. Results

### 3.1. Experimental Results

#### 3.1.1. Screening for Plant Extracts That Enhance Cellular Reprogramming

**Figure 1:**
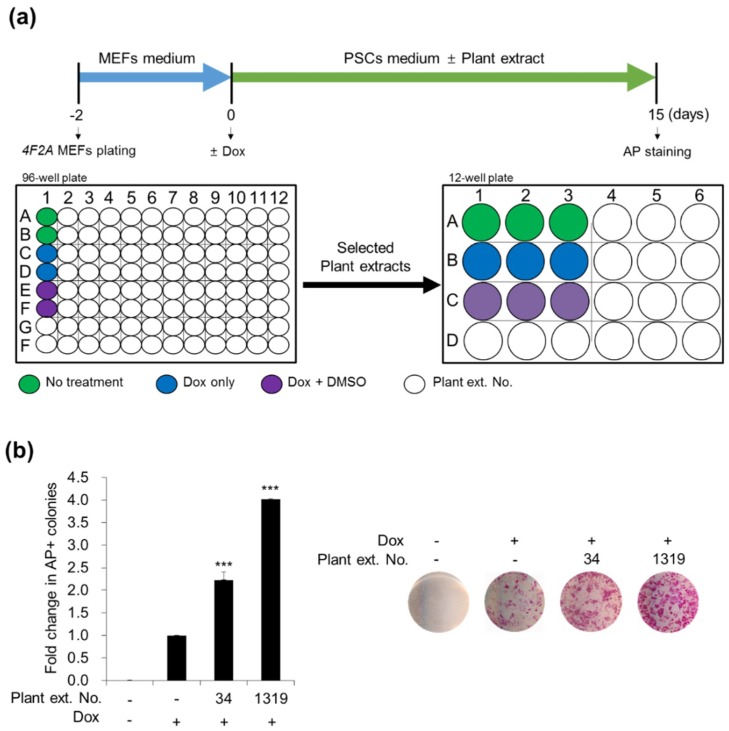
Screening and identification of plant extracts No. 34 and No. 1319 from *Camellia japonica* (*CJ*) that promote mouse embryonic fibroblasts (MEF) reprogramming. (**a**) Schematic experimental procedure of high-throughput screening. The *4F2A* MEFs were seeded at 7.5 × 10^3^ cells per well in 96-well plates. After 2 days, cells were treated with or without plant extracts and cultured in doxycycline (dox) to activate Oct-4, Sox-2, Klf-4, and c-Myc (OSKM) expression. The number of alkaline phosphatase (AP)+ colonies on day 15 post-treatment was used as a measure of the reprogramming efficiency. (**b**) Identification of *CJ* extracts No. 34 and No. 1319 that enhance reprogramming. The relative fold changes of AP+ colonies compared with dimethyl sulfoxide (DMSO)-treated control and cultured in dox were presented as the mean + standard error of the mean (SEM) of three independent experiments. Statistical significance was determined using Student’s *t*-test (*** *p*-value < 0.001). Representative images of AP+ colonies per well were presented in the right panel.

To investigate whether plant extracts could enhance cellular reprogramming, we used *4F2A* MEFs to screen for their reprogramming efficiency. The reprogramming scheme we used in this work is consistent with the published procedures [47,48] with modifications (Figure 1a). As previously reported [48], *4F2A* MEFs can generate mouse iPSCs by culture in dox. Cells were treated with or without crude plant extracts at a final concentration of 25 µg/mL. Subsequently, we evaluated cellular reprogramming efficiency by counting the number of alkaline phosphatase (AP)-positive mouse embryonic stem cell (ESC)-like colonies 15 days later (Figure 1a). We obtained AP-positive ESC-like colonies only when dox was added and selected an initial list of different plant extracts which could affect to reprogramming efficiency (Figure 1a). Effects of other extracts (for example, extracts from *Ilex integra*, *Quercus gilva*, *Celtis choseniana*, *Staphylea bumalda*, *Ligustrum japonicum*, *Daphniphyllum macropodum*, *Litsea japonica*, *Cinnamomum camphora*, *Alnus maximowiczii*, *Styrax japonica*, *Zanthoxylum ailanthoides*, and so on) from the primary screening were not significant. Among the selected extracts, No. 34 and No. 1319 were demonstrated to increase the number of AP-positive ESC-like colonies by ~2.23-fold (2.23 ± 0.16) and ~4.01-fold (4.01 ± 0.23), respectively, compared with control DMSO treatment (Figure 1b). Interestingly, both No. 34 and No. 1319 were extracted from *CJ*. No. 34 was obtained from stems, and No. 1319 was obtained from fruits. Moreover, *CJ* flower extracts also induced an approximately 2-fold (2.31–2.52-fold) increase in reprogramming efficiency compared with control DMSO treatment. These results suggest that crude extracts of *CJ* plant may accelerate the somatic cell reprogramming efficiency of MEFs.

#### 3.1.2. Stimulatory Effects of *CJ* Extracts on Human cell Reprogramming 

**Figure 2:**
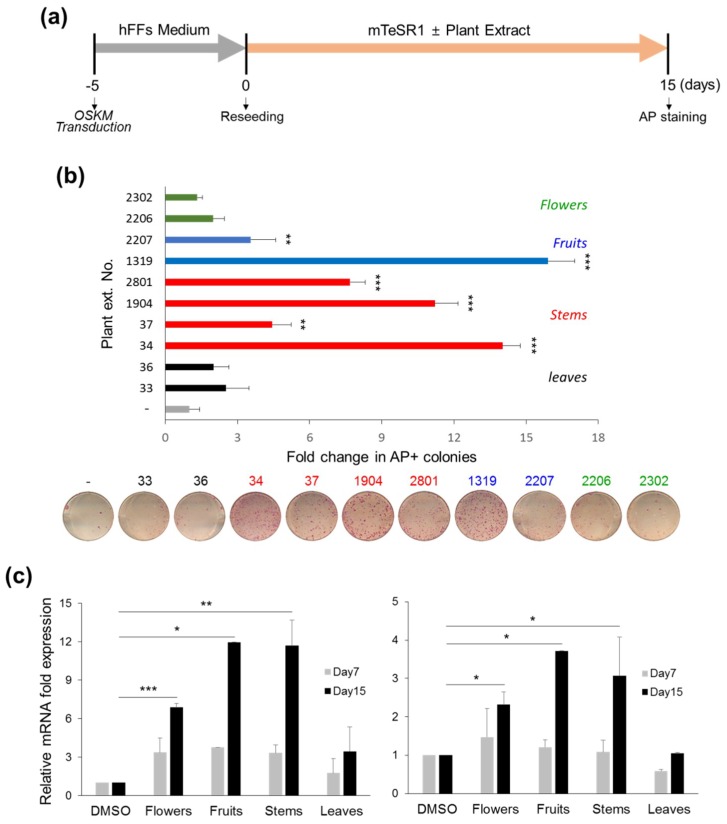
Identification of extracts from fruit and stem of *CJ* enhance human somatic cell reprogramming. (**a**) Schematic experimental procedure of human pluripotent stem cell (hiPSC) generation. The human foreskin fibroblasts (hFFs) were seeded at 1 × 10^5^ cells per well in six-well plates, and cells were transduced with OSKM-Sendai virus (SeV) on the following day. After five days, OSKM-transduced hFFs were reseeded on Matrigel-coated 12-well plates and treated with or without different *CJ* extracts. (**b**) The groups of crude extracts from fruits or stems of *CJ* promote hiPSC generation. The number of AP+ colonies on day 15 post-treatment was used as a measure of the reprogramming efficiency. The relative fold changes in AP+ colonies compared with untreated controls (designated as 1) are presented. (**c**) The mRNA expression of the pluripotency markers Rex1 (left) and Nanog (right) at reprogramming days 0, 7, and 15 by qPCR. The results are presented as the mean + SEM (*n* = 3). The statistical significance was determined using Student’s *t*-test (* *p* < 0.05, ** *p* < 0.01, *** *p* < 0.001).

To test whether *CJ* extracts are involved in human somatic cell reprogramming, we transduced human foreskin fibroblasts (hFFs) with Sendai virus (SeV) vectors encoding Oct4, Sox2, Klf4 and L-Myc for transgene-free generation of hiPSCs following the reprogramming scheme (Figure 2a). Five days after viral infection, transduced hFFs were reseeded and treated with or without each 10 different types of *CJ* plant extracts from flowers (Nos. 2302 and 2206), fruits (Nos. 2207 and 1319), stems (Nos. 2801, 1904, 37, and 34), and leaves (Nos. 36 and 33) in mTeSR1 growth medium at a final concentration of 25 µg/mL. As expected, we obtained AP-positive, human ESC (hESC)-like colonies from OSKM-SeV transduced hFFs 15 days after treatment. We observed that *CJ* extract treatment, including extracts from flowers (No. 2302: 1.33 ± 0.21 and No. 2206: 1.99 ± 0.46), fruits (No. 2207: 3.56 ± 1.02 and No. 1319: 15.91 ± 1.12), stems (No. 2801: 7.68 ± 0.64, No. 1904: 11.21 ± 0.97, No. 37: 4.44 ± 0.79, and No. 34: 14.03 ± 0.73), and leaves (No. 36: 2.01 ± 0.64 and No. 33: 2.53 ± 0.95) enhanced the number of AP-positive colonies compared with control DMSO treatment (Figure 2b). Among these extracts, the extracts obtained from fruits and stems exhibited significant reprogramming stimulatory effects (Figure 2b, fruits: blue bars, average 9.73-fold; stems: red bars, average 9.34-fold). Similar results were verified by analyzing the expression level of the pluripotency markers, such as Oct4, Rex1 (Figure 2c left, fruits: average 11.97-fold; stems: average 11.70-fold), and Nanog (Figure 2c right, fruits: average 3.72-fold; stems: average 3.06-fold) at reprogramming day 15. Interestingly, No. 1319 and No. 34 most effectively induced human somatic cell reprogramming, inducing levels similar to those observed for mouse reprogramming using *4F2A* MEFs. These data suggest that No. 1319 and No. 34 *CJ* extracts function in efficient reprogramming in both human and mouse somatic cells.

#### 3.1.3. Synergistic Effects of *CJ* Extracts from Fruit and Stem on Human Cell Reprogramming 

**Figure 3:**
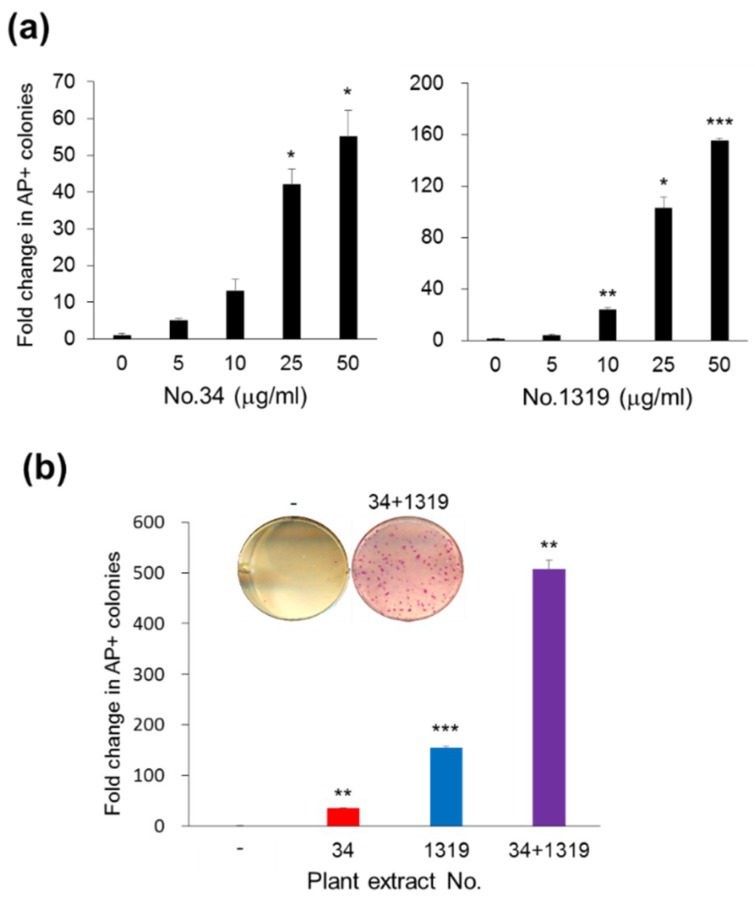
Synergistic effects of fruit and stem extracts of *CJ* on hiPSC generation. (**a**) Dose-dependent enhancement of reprogramming efficiency by *CJ* extracts (No. 34: stem and No. 1319: fruit). OSKM-transduced hFFs were incubated with or without *CJ* extracts at the indicated concentrations. (**b**) *CJ* stem and fruit extracts exhibited synergistic effects on stimulated reprogramming with each other. hFFs were transduced by OSKM-SeV and cultured with No. 34, No. 1319 (50 µg/mL), and the combination of No. 34 and No. 1319 (in half at a final concentration of 50 µg/mL). The human ESC (hESC)-like colonies were stained for AP and quantified to determine reprogramming efficiency. The relative fold changes in AP+ colonies compared with untreated controls (designated as 1) are presented. The results are presented as the mean + SEM (*n* = 3). Statistical significance was determined using Student’s *t*-test (* *p* < 0.05, ** *p* < 0.01, *** *p* < 0.001).

To further determine the effective concentration of No. 34 or No. 1319 for human cell reprogramming, we treated OSKM-SeV transduced hFFs with various concentrations of No. 34 or No. 1319. AP-positive colonies with ESC-like morphology derived from hFFs were assessed after 15 days. As shown in Figure 3a, No. 34 or No. 1319 treatment enhanced AP-positive colony yield in a dose-dependent manner up to 50 µg/mL. These results indicate that 50 µg/mL of No. 34 or No. 1319 is an effective concentration for hiPSC generation (Figure 3a). Increasing the concentration of extracts up to 100 µg/mL did not enhance reprogramming efficiency. Interestingly, the combination of No. 34 and No. 1319 at a final concentration of 50 µg/mL exhibited significantly increased reprogramming enhancement by approximately 508-fold compared with control DMSO treatment compared with either extract alone at the same concentration (Figure 3b). These results demonstrate that the combination of No. 34 and No. 1319 *CJ* extracts from stem and fruit improves human iPSC generation.

#### 3.1.4. Characterization of the *CJ*-Extract-Induced hiPSC Pluripotency Ability

**Figure 4:**
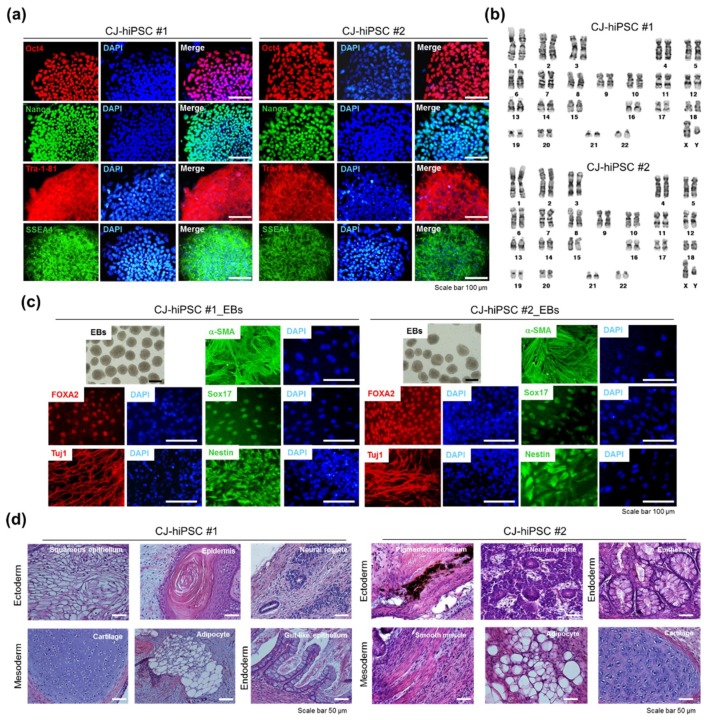
Characterization of *CJ*-extract-induced hiPSC clones (*CJ*-hiPSCs). (**a**) Representative images of *CJ*-hiPSCs#1 and #2 depicting typical morphology and expression of pluripotency markers, including Oct4, Nanog, SSEA-4, and Tra-1-81. Scale bars, 100 µm. (**b**) Normal karyotype of *CJ*-hiPSCs#1 and #2. (**c**) In vitro embryoid body (EB) differentiation of *CJ*-hiPSCs#1 and #2. Bright field images of representative EBs and immunocytochemical analysis of mesoderm (a-SMA), endoderm (FOXA2 and Sox17), and ectoderm (Tuj1 and Nestin) markers in differentiated cells. The cells were co-stained with DAPI (blue). Scale bars, 100 µm. (**d**) Histological analysis of teratomas derived from *CJ*-hiPSCs#1 and #2. Ectoderm was represented by neural tissue and epidermis. Mesoderm was represented by cartilage, adipocyte, and muscle. Endoderm was represented by epithelium. Scale bars, 50 µm.

Next, we randomly isolated two hiPSC colonies derived from reprogramming by No. 34 and No. 1319 treatment for 3 weeks based on their hESC-like morphology for further characterization of pluripotency. These hiPSC clones were expanded in mTeSR1 growth media without *CJ* extracts. The *CJ*-extract-induced hiPSC clones (*CJ*-hiPSC#1 and *CJ*-hiPSC#2) were morphologically similar to normal hESCs and expressed high levels of pluripotency markers (Nanog, Oct4, TRA-1-81, TRA-1-60, SSEA3, and SSEA4) as determined by immunofluorescence staining (Figure 4a). In vitro differentiation potential of *CJ*-hiPSCs was demonstrated by the formation of embryoid bodies (EBs) in suspension culture and subsequent EB differentiation. All three embryonic germ layers were revealed by immunofluorescence staining with specific markers for the ectoderm (anti-Tuj1 and anti-Nestin), mesoderm (anti-Desmin and anti-α-SMA), and endoderm (anti-Sox17 and anti-Foxa2) (Figure 4b). We also confirmed a normal karyotype (Figure 4c) and the in vivo pluripotency (Figure 4d) of *CJ*-hiPSC clones. Histological examination revealed that *CJ*-hiPSCs contributed to all three embryonic germ layers (Figure 4d). Taken together, these data indicate that *CJ* extract treatment induces sustainable hiPSC features of pluripotency.

#### 3.1.5. Positive Effects of *CJ* Extracts on Re-Epithelialization in Wound Healing

**Figure 5:**
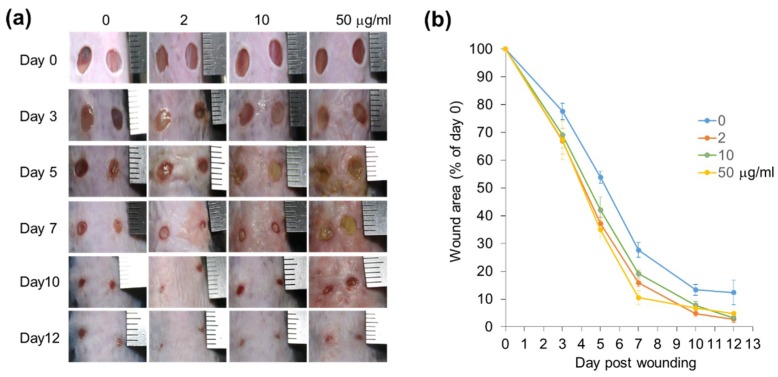
Effects of *CJ* extracts from fruit and stem on the wound healing process. (**a**) Rapid wound closure was observed in *CJ*-extract-treated wounds. Wounds treated with the indicated concentrations of extracts on days 0, 3, 5, 7, 10, and 12 were presented. (**b**) Wound areas in control and treated mice were determined by tracing the wound margin using a transparent sheet. The results were presented as the mean ± SEM (*n* = 8) for each time point and group.

To examine regenerative effects of *CJ* extracts No. 34 and No. 1319, we used the in vivo mouse wound healing model for skin regeneration. To assess the efficacy of *CJ* extracts, wound size was observed after treatment with *CJ* extracts at 2, 10, and 50 mg/mL for 12 days. On the first day of the experiment, all wounds were of similar size and exhibited a red color (Figure 5a). On day 3 postwounding, a brown red clot covering the wounds of the *CJ*-extract-treated groups was observed (Figure 5a). This finding was indicative of scab formation and initiation of the wound healing process. These clots were significantly observed when wounds were treated with 10 or 50 mg/mL *CJ* extract for 5 days compared with untreated control (Figure 5a). No significant differences were noted between treated and untreated DMSO as previously reported [51]. The wounds treated with *CJ* extracts, including wounds treated with 2 mg/mL *CJ* extracts, became more homogeneous and consistent in texture on day 10 (Figure 5a). In addition, the size of the wound area was significantly reduced in all groups treated with *CJ* extracts (2, 10, and 50 mg/mL) from day 3 compared with control wounds (Figure 5a,b). The wound closure rates were more rapidly improved in all *CJ* extract treatment groups compared with the control group. In particular, on day 7, the wound areas treated with *CJ* extracts at concentrations of 2 mg/mL (~16% of original wound area) and 50 mg/mL (~11% of original wound area) were more closed on average by 1.7- and 2.6-fold, respectively, compared with the control (~28% of original wound area) (Figure 5b).

**Figure 6:**
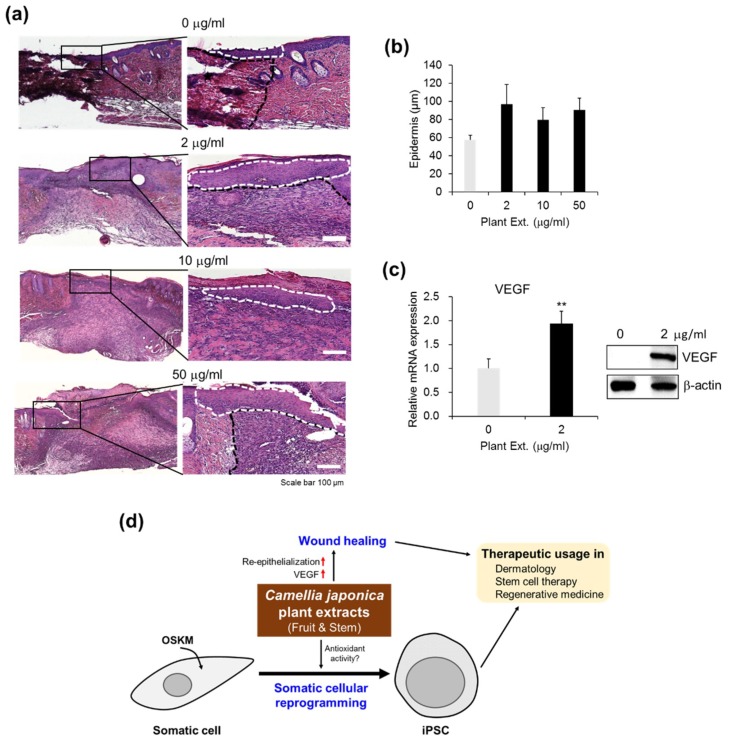
Re-epithelialization and vascular endothelial growth factor (VEGF) upregulation by *CJ* extracts in wound healing. (**a**) Histological examination of mice treated with *CJ* extracts No. 34 and No. 1319 (0–50 mg/mL) on the day 7 after wounding. The photographs are representative of three wounds stained with hematoxylin and eosin in each group. Dotted lines denote the route of re-epithelialization. (**b**) The length of the epidermis based on re-epithelialization from the wound edges was analyzed histomorphometrically. The results are presented as the mean ± SEM (*n* = 8). (**c**) VEGF expression in the wound tissues on day 7 postsurgery was quantified by qPCR analysis (left) and by Western blot analysis (right). VEGF mRNA expression was normalized to GAPDH. The results are presented as mean ± SEM (*n* = 4) and ** *p* < 0.01. (**d**) Putative model of *CJ*-extract-mediated reprogramming and wound healing.

Next, we investigated whether *CJ* extracts affect re-epithelialization at the wound sites based on histological analysis of *CJ*-extract-treated wound tissues. To observe the change in epidermal thickness, wound sites were treated with *CJ* extracts (0–50 mg/mL) every other day. Seven days after treatment, epidermal thickness and length were significantly increased in the *CJ*-extract-treated groups compared to the control (Figure 6a,b), indicating that *CJ* extracts are effective in re-epithelialization at the wound sites. In addition, the lowest concentration of *CJ* extracts (2 mg/mL) was sufficient to enhance wound healing (Figure 6a,b). Taken together, these results suggest that *CJ* extracts enhance cutaneous wound healing in mice. 

#### 3.1.6. Upregulation of VEGF Expression by *CJ* Extracts Promotes Wound Healing

Wound healing is a dynamic process that consists of three phases: tissue inflammation, proliferation, and remodeling. These phases are regulated by different cytokines and growth factors that are secreted by inflammatory or resident cells [52,53]. In particular, VEGF is a critical angiogenic factor in the chronic inflammation model and stimulates wound angiogenesis and endothelial cell migration and proliferation in a paracrine manner [54,55,56]. Compared with control wounds, wound tissues treated with *CJ* extracts No. 34 and No. 1319 at a concentration of 2 mg/mL increased the VEGF mRNA level (Figure 6c, left) as well as the protein level (Figure 6c, right). These results indicate that *CJ* extracts from fruit and stem are involved in wound healing, improving vascular function via VEGF upregulation. 

## 4. Discussion

We report here that *CJ* extracts potentially enhance the acquisition of pluripotency in vitro and wound healing in vivo in a mouse model. We demonstrated that *CJ* extracts of the fruit and/or stem exhibited a positive effect on reprogramming somatic cells to pluripotency using a high-throughput screening strategy of 3695 plant extracts in genetically homogeneous secondary MEFs harboring the dox-inducible reprogramming transgenes (OSKM). We demonstrated that a combination of the extracts from *CJ* fruit and stem significantly improved hiPSC generation from human skin cells and promoted efficient wound healing by improving re-epithelialization and VEGF expression at the wound sites. These findings suggest that clinical-grade pharmacological approaches to improve reprogramming efficiency and tissue regeneration are concurrently applicable to stem-cell-related regenerative medicine (Figure 6d). 

By simply adding *CJ* extracts to the culture medium during the entire reprogramming process, we obtained high-quality iPSCs from both mouse and human cells. Among the several different parts of *CJ* investigated in the present study, the most active extract was No. 1319 obtained from the whole fruit of *CJ* (15.91-fold increase) (Figure 2). An extract of *CJ* fruit rind (No. 2207) was also effective in hiPSC generation but less potent (3.5-fold increase). Similarly, *CJ* stem extracts, including No. 34 (whole stem), Nos. 1904 and 2801 (the extracts of stem bark), and No. 37 (the extracts of stem heartwood), promoted hiPSC generation. Among these extracts, No. 34 was most effective (14.03-fold increase) (Figure 2). Our results suggest that the whole extracts from fruit and stem of *CJ* can be used for pluripotency control.

During pluripotent reprogramming, OSKM-transduced cells exhibit substantially elevated reactive oxygen species (ROS) levels and oxidative stress [57,58]. ROS are tightly regulated by ROS scavengers, such as antioxidant enzymes, under normal physiological conditions. However, excessive accumulation of ROS occurs in certain conditions, and cells experience difficulties in detoxifying ROS when present at levels beyond the capacity of the antioxidant cellular defense system [59,60]. Increased ROS levels lead to cell damage, senescence, and interference in generation and maintenance of iPSCs [61,62]. Recent studies demonstrated that the addition of antioxidants, such as N-acetyl-cysteine and vitamin C (Vc), prevents this damage and subsequently improves iPSC generation with a significant reduction in de novo copy number variations (CNVs) [9,63]. *CJ* extracts exhibit a variety of biological activities, such as antioxidant [33,34] and antiaging activities [41]. Thus, *CJ*-extract-mediated reprogramming promotion is potentially associated with antioxidant activity.

Vc has also been suggested to restore the activity of collagen prolyl hydroxylases that regulate collagen synthesis to maintain tissue integrity and efficient wound healing [63,64]. Similarly, *CJ* extracts not only exhibit reprogramming-promoting activity but also effectively impact wound healing (Figure 5 and Figure 6). The beneficial effects of plant extracts on wound healing has been supported by several experimental studies [65]. Similarly, oil from *CJ* seeds induce human type I procollagen synthesis and inhibit matrix metalloproteinase (MMP)-1 activity, suggesting involvement in skin barrier function and aging [41]. Therefore, we suggest that *CJ* extracts with antioxidant activity are involved in somatic cellular reprogramming to pluripotency, re-epithelialization, and effective wound healing. As bioactive constituents of *CJ*, oleanane triterpenes from the extracts of *CJ* fruits peels exhibit strong protein tyrosine phosphatase 1B (PTP1B) inhibitory activity and cytotoxicity against several human breast cancer cell lines [66]. Triterpenoids from stem bark extracts of *CJ* also exhibit cytotoxicity against cancer cell lines [67]. Moreover, the isolated compounds 28-Nor-oleanane-type triterpene saponins from stem bark extracts of *CJ* exhibit inhibitory activity on NO production in macrophages and might serve as an anti-inflammatory agent [68]. However, the potential bioactive constituents and their molecular mechanism involved in pluripotency remain largely unknown.

Research on pharmacological approaches for stem cell fate transition and regeneration is important for stem-cell-related regenerative medicine and therapy. Clinically, the generation of genetically unmodified hiPSCs, restoration of tissue integrity, and efficient wound healing are critical to alleviate stress. Natural plant extracts from the fruit and stem of *CJ* will potentially contribute to the development of the field of regenerative medicine and function as novel natural enhancers of somatic cellular reprogramming and wound healing.

## 5. Conclusions

In conclusion, these studies suggest that natural plant products from *CJ* fruit and stem exhibit positive biochemical and/or histopathological effects on in vitro somatic reprogramming into pluripotency and in vivo wound healing in a mouse model. Based on these findings, we suggest that *CJ* extracts potentially represent useful supplements for the generation of clinical-grade hiPSC and/or direct treatment for skin diseases. However, further studies that investigate the identification and efficacy of bioactive constituents from *CJ* fruit and stem extracts are required prior to the clinical application of these extracts.
